# Workout Duration Alters the Importance of Predictive Traits on High-Intensity Functional Training Workout Performance

**DOI:** 10.3390/sports13060156

**Published:** 2025-05-22

**Authors:** Gerald T. Mangine, Kristyn C. McGeehan, Wil King, Ashley Hines, James W. Henley, Jacob L. Grazer, Tiffany A. Esmat, John R. McLester

**Affiliations:** Department of Exercise Science and Sport Management, Kennesaw State University, Kennesaw, GA 30144, USA; kmcgeeh2@students.kennesaw.edu (K.C.M.); wking39@students.kennesaw.edu (W.K.); ahines19@students.kennesaw.edu (A.H.); jhenle16@students.kennesaw.edu (J.W.H.); jgrazera@kennesaw.edu (J.L.G.); tesmat@kennesaw.edu (T.A.E.); jmcleste@kennesaw.edu (J.R.M.)

**Keywords:** CrossFit^®^, HIFT, programming, repetition completion rate, volume load, kinetics, velocity, power

## Abstract

Relevant skills and physiological traits vary between high-intensity functional training (HIFT) workouts, but it is unknown how altering each programming detail affects their importance. To examine the effect of workout duration on relationships to HIFT performance, twelve men and ten women with ≥2 years of HIFT experience (29.3 ± 7.1 years, 171 ± 7 cm, 80.5 ± 15.6 kg) completed a baseline visit to assess body composition and performance in vertical jump, barbell thruster, and 2000 m rowing tests. Participants returned twice to randomly complete the same circuit of rowing, barbell thrusters, and box jumps for “as many repetitions as possible” in 5 or 15 min. Performance was described by expressed kinetics on each exercise, overall and individual exercises, repetition completion rates and volume load completed, and transition times. Spearman correlation analysis revealed several expected relationships (*p* < 0.05) but also differences between workout durations. Performance measures, especially rowing (time: ρ = −0.89 to −0.94; power: ρ = 0.88 to 0.93), were more applicable to the 5 min workout. Experience and body composition measures (ρ = −0.47 to −0.50) were more often related to the 15 min transition strategy. These data suggest that increasing workout duration alters the importance of relevant predictive traits.

## 1. Introduction

Athletes prepare for the unique physical demands of their sport by utilizing sports-specific training regimens to stimulate adaptations in relevant physiological traits [1,2,3]. This is a relatively straightforward process for novice trainees and athletes who compete in most traditional sports. Training generally follows a linear path that promotes peak strength and power during the competitive season because these traits often underpin athletic success [1,4]. However, this process is more complicated for experienced athletes and those who compete in sports that require simultaneous aptitude in several competing physiological traits (e.g., tactical or CrossFit^®^ athletes). Greater attention to prescription and the organization of workout sessions is needed to ensure that training addresses relevant traits without being redundant and overstressing the athlete [1,2,3]. These are inherent risks of high-intensity functional training (HIFT; e.g., CrossFit^®^), which aims to promote simultaneous adaptations across multiple fitness domains through daily variation in workout design [5,6]. Failing to account for the differential effects of training competing physiological systems could elevate the risk of overtraining and injury.

Risks can be reduced through purposeful prescription and monitoring training loads, though there are no commonly accepted methods for equating HIFT workouts [7]. Individual workouts are often structured into circuits that are either completed for a set number of times as quickly as possible, or the circuits are repeated for “as many repetitions as possible” [AMRAP] within a set time limit. Circuits may be composed of a wide variety of exercises, with each potentially being given a different intensity and volume prescription. Since intensity and work are quantified differently across modalities, it is hard to describe how workout combinations are interrelated. Strong relationships amongst different HIFT workouts have been reported on multiple occasions [8,9,10], but their associations with various athletic characteristics (e.g., experience, body composition, strength, aerobic and anaerobic fitness, etc.) have not been in consensus [8,9,11,12,13,14,15,16,17]. There does not appear to be a hierarchy amongst traits that would allow those that are most indicative of performance to be identified, and thus, most deserving of attention during training. This is partially the consequence of studies not being consistent in their definition of “experience” (0–24+ months, competition rankings), and most limit their examination to only one or a few predictors of performance in no more than a handful of workouts. Without anything to tie workouts together, the observed relationships cannot be generalized. There are simply too many uncontrolled factors.

It is reasonable to assume that requisite physiological traits and skills will differ between HIFT workouts. However, little is known about how much needs to be different to completely change the stimulus. Comparison studies have shown differential physiological responses [6,18], but these have always been between workouts that shared very few commonalities. This makes it impossible to determine which programming detail(s) is/are responsible for any observed differences. To the best of our knowledge, only one study has attempted to compare near-identical workouts [19]. Toledo and colleagues (2021) had participants complete a 20 min AMRAP circuit and recorded heart rate, lactate, and perceived exertion responses. Participants returned after 72 h to complete a second workout that adjusted repetition assignments so that the same volume of work could be completed in a “rounds for time” structure. The adjustments led to a shorter workout duration (by approximately 1.1 min) and greater lactate response (+20.6%) in men only. No other differences were seen. While this study is a step in the right direction, it still leaves too many programming details uncontrolled. The exercises and total volume of work remained the same, but repetitions, workout duration (in men only), and rounds completed were all different. Any of these factors might have been responsible for the enhanced lactate response seen in men. Thus, the purpose of this study was to examine the effect of altering only a single prescription detail (i.e., workout duration) of an AMRAP circuit on relationships between workout performance and athlete experience, body composition, and performance measures.

## 2. Materials and Methods

### 2.1. Participants

Data for this study were drawn from a larger investigation about the effects of a multi-ingredient pre-workout supplement on HIFT performance [20]. That study was approved by the University Institutional Review Board (#IRB-FY23-18) and recruited twenty-two men and women with at least two years of HIFT experience but had never reached the advanced stages of any international HIFT competition. Participants were recruited via word of mouth and flyers shared at local training facilities and competitions throughout the Metro Atlanta area. Each participant was verified to be free from any injury or illness that could have impacted their performance and was not using any performance-enhancing drugs or medications (as determined by PAR-Q+ and Health and Medical History Questionnaire) that would have invalidated their performance in official competition before providing their written informed consent to participate. Participant baseline characteristics are presented in Table 1.

### 2.2. Study Design

**Figure 1:**
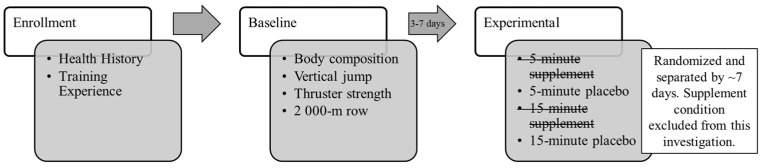
Study design.

The larger investigation had participants report to the Human Performance Laboratory on five separate occasions within a 5–6-week period (see Figure 1). Data related to each participant’s training history (e.g., age, years of resistance training, gymnastics, and HIFT experience) were collected during enrollment. Body composition, vertical jump height, barbell thruster strength, and 2000 m rowing time were tested on their baseline visit (i.e., the first visit). All remaining visits were devoted to experimental workout sessions that were completed in a randomized, cross-over fashion. Experimental visits had participants complete either a 5 or 15 min AMRAP workout 40 min after having consumed a multi-ingredient pre-workout supplement or placebo. All workouts were video recorded and analyzed to obtain pacing data about the overall workout and each individual component (e.g., exercises and transitions). Expressed kinetics during each exercise were obtained via the rowing ergometer microcomputer, a portable three-dimensional motion-tracking camera, or in-ground force plates. All visits occurred on the same day of the week at a time of day that was consistent with each participant’s normal workout time. The present study related data collected on the baseline visit with pacing and kinetics expressed during the placebo workouts only. The relationships with the 5 and 15 min AMRAPs were then compared to determine how workout duration affected relevant characteristics.

### 2.3. Baseline Testing

Participants began testing with an initial measurement of height (±0.1 cm) and body mass (±0.1 kg) while wearing athletic attire and standing barefoot on a stadiometer (WB-3000, TANITA Corporation, Tokyo, Japan). Subsequently, a 4-compartment model was used to estimate body fat percentage (BF%) and fat-free mass (±0.1 kg) using standard procedures for three common methods of assessment [21,22]. The model used bone mineral content estimated from dual-energy X-ray absorptiometry (Lunar Corporation, Madison, WI, USA), body volume from air displacement plethysmography (BodPod, COSMED USA Inc., Chicago, IL, USA), and total body water from bioelectrical impedance analysis (770 Body Composition and Body Water Analyzer, InBody, Seoul, Republic of Korea).

Afterwards, participants began a standardized warmup and then completed performance testing in the following order: vertical jump height, barbell thruster strength, and 2000 m rowing for time. All performance tests were completed in the presence of a certified strength and conditioning specialist (CSCS). Verbal encouragement was given on all maximal tests. Vertical jump height was performed using a testing station (Vertec, JUMPUSA, Sunnyvale, CA, USA) and calculated as the difference between maximal jump height and maximal vertical reach in inches and then converted to meters (m). Mean vertical jump power (in Watts) was estimated via the Harman formula [23]. Barbell thruster strength was estimated from a 3–5 repetition maximum (RM) thruster assessment.

Participants were allotted 1–2 maximal trials to find the highest load they could complete 3–5 repetitions of the barbell thruster exercise while maintaining the technical standards employed in official HIFT competition [24]. Five minutes of rest were allotted if a second trial was deemed necessary. One-repetition maximum was estimated via the Brzycki estimation formula [25], and relative strength was calculated by dividing the estimate by the participant’s body mass.

The 2000 m rowing test was completed on a rowing ergometer (model D, Concept2 Inc, Morrisville, VE, USA) using its microcomputer to set a 2000 m countdown to initiate starting with the participant’s first pull. The test concluded, and time (in minutes) and average power (in Watts) were automatically recorded when the counter reached zero meters.

### 2.4. Workout Protocol and Kinetics Assessments

Participants initiated all experimental workout sessions with a standardized warmup, rested for five minutes, and then completed either a 5 or 15 min workout protocol. Regardless of duration, the workout consisted of the same AMRAP circuit of rowing on an ergometer (9 Calories per round for men, 7 Calories per round for women), six barbell thrusters (43.1 kg for men, and 29.5 kg for women), and three box jumps to a standard height (0.61 m for men and 0.51 m for women) with mandatory step down. These exercises and their prescription were selected because they commonly appear in HIFT [24,26,27] and enabled the collection of movement kinetics using our laboratory’s available technology. These sessions were supervised by a CSCS and verified by video recordings collected by a 3.5-megapixel Microsoft Surface 3 tablet camera (Microsminoft Corp., Redmond, WA, USA) at 1920 × 1080 pixels/30 frames per second using previously described positioning standards [28]. Performances on both workouts are presented in Table 2.

The methods used to quantify all pacing and expressed kinetics data have been described in detail elsewhere [28]. Video recordings were analyzed to verify performance scores and quantify pacing variables of interest. Workouts were scored as total rounds and repetitions completed and converted to round and repetition completion rate (per minute) and total volume load completed (i.e., sum of rowing, thruster, and box jump volume loads). Additionally, videos were analyzed to calculate the average repetitions completion rate (i.e., repetitions [or Calories] per second) for each exercise, average time spent transitioning between exercises, and a coefficient of variation (CV; standard deviation across rounds divided by the average) for average transition time across rounds [29,30,31].

Three different technologies were used to monitor expressed kinetics during each workout. Data retained by the rowing ergometer microcomputer were transferred via a flash drive to the Concept2 logbook application and used to calculate average strokes per minute and power (in Watts) expressed across rounds. Total rowing workload (in kg) was derived from the product of average power, total rowing strokes, and total rowing duration divided by total distance covered. Mean barbell velocity (m·sec^−1^) and power (Watts) expressed across repetitions and rounds were monitored by a three-dimensional motion-tracking camera (PERCH, Catalyft Labs, Inc., Cambridge, MA, USA). Forces expressed onto the ground during box jumps were measured by a pair of in-ground connected force plates (BMS400600 plates, © 2024 Advanced Mechanical Technology, Inc., Watertown, MA, USA) sampling at a rate of 1000 Hz. Jumping data were analyzed by a custom software application developed in LabView (v. Q3, 2023, National Instruments, Austin, TX, USA) to produce mean force (N), rate of force development (RFD; N·sec^−1^), and impulse (N·sec) for the 2nd and 3rd jumps of each set, which were then averaged for analysis. The first jump was discarded due to variability from its unique starting position compared to subsequent jumps. Box jumps volume load was calculated as the product of total repetitions and mean force.

### 2.5. Statistical Analysis

The results of the Shapiro–Wilk test indicated that some variables of interest were not normally distributed. Therefore, relationships between baseline and workout performance variables were evaluated by Spearman’s rho (*ρ*) correlation coefficients. The strength of the correlations was interpreted using the following criteria: trivial (<0.10), small (0.10–0.29), moderate (0.30–0.49), high (0.50–0.69), very high (0.70–0.90), or practically perfect (>0.90) [32]. Comparisons between relationships to 5 and 15 min workout performances were then made by applying Fisher’s z-transformation to each correlation coefficient to convert them into z-scores and then comparing z-scores with paired samples *t*-tests [33]. All statistical analyses were performed using SPSS (v. 29, Chicago, IL, USA) with a criterion alpha set at *p* ≤ 0.05. All data are reported as mean ± standard deviation.

## 3. Results

### 3.1. Age and Training Experience

Age was positively related to a 15 min CV of barbell thruster transition time (*p* = 0.026), but this was not significantly different from 5 min. No relationships were found between resistance training experience and workout performance.

Gymnastics experience was negatively related to average transition time (overall and when transitioning to box jumps; *ρ* = −0.48 to −0.50, *p* < 0.05) and the CV of transition time to thrusters (*ρ* = −0.46, *p* = 0.038) during the 15 min workout but not the 5 min workout. The relationship to the CV of transition time to thrusters was significantly different from the 5 min bout (*ρ* difference = 0.22, z = 2.16, *p* = 0.031). A positive correlation was seen with box jump repetition completion rate of the 15 min workout (*ρ* = 0.50, *p* = 0.020), but this was not significantly different from the 5 min workout. HIFT experience was positively related to average transition time (overall and when transitioning to rowing; *ρ* = −0.49 to 0.57, *p* < 0.05) and thruster repetition completion rate (*ρ* = 0.45, *p* = 0.046) during the 5 min workout, and these relationships (except for thruster repetition completion rate) were significantly different from the 15 min workout (*ρ* difference = 0.46 to 0.48, z = 2.15, *p* = 0.031). A negative relationship was seen with a 15 min CV of transition time to box jumps (*ρ* = −0.45, *p* = 0.048), but this was not significantly different from the 5 min workout. Other significantly different (*p* < 0.05) relationships were noted, but they included variables that were not significantly related to either 5 or 15 min workout performance. The differences amongst relationships between workout performance and age and training experience are presented in Table 3.

### 3.2. Body Composition

The differences amongst relationships between workout performances and body composition are illustrated in Table 4. The positive relationships between fat-free mass and round completion rate (*ρ* = 0.45, *p* = 0.037), and between height and rowing volume load (ρ = 0.63, *p* = 0.002), were only seen with the 5 min workout. Additionally, though height, body mass, and fat-free mass were all positively related to rowing power during both durations (*ρ* = 0.53 to 0.88, *p* < 0.05), stronger correlations were seen with the 5 min workout (*ρ* difference = 0.24 to 0.26, z = 1.98 to 2.39, *p* < 0.05).

Negative relationships between the CV of transition times and body mass (*ρ* = −0.47, *p* = 0.034), the CV of transition times and fat-free mass (*ρ* = −0.50, *p* = 0.022), and fat mass and body fat percentage and box jump repetition completion rate (*ρ* = −0.53 to −0.54, *p* < 0.05) were only seen with the 15 min workout. The positive relationships between height and fat-free mass and transition times to rowing (*ρ* = 0.58 to 0.61, *p* = 0.003) were also only seen with the 15 min workout. Additionally, height had a stronger relationship to thruster power during the 15 min workout (*ρ* difference = 0.07, z = 2.29, *p* = 0.022).

### 3.3. Performance Measures

**Figure 2:**
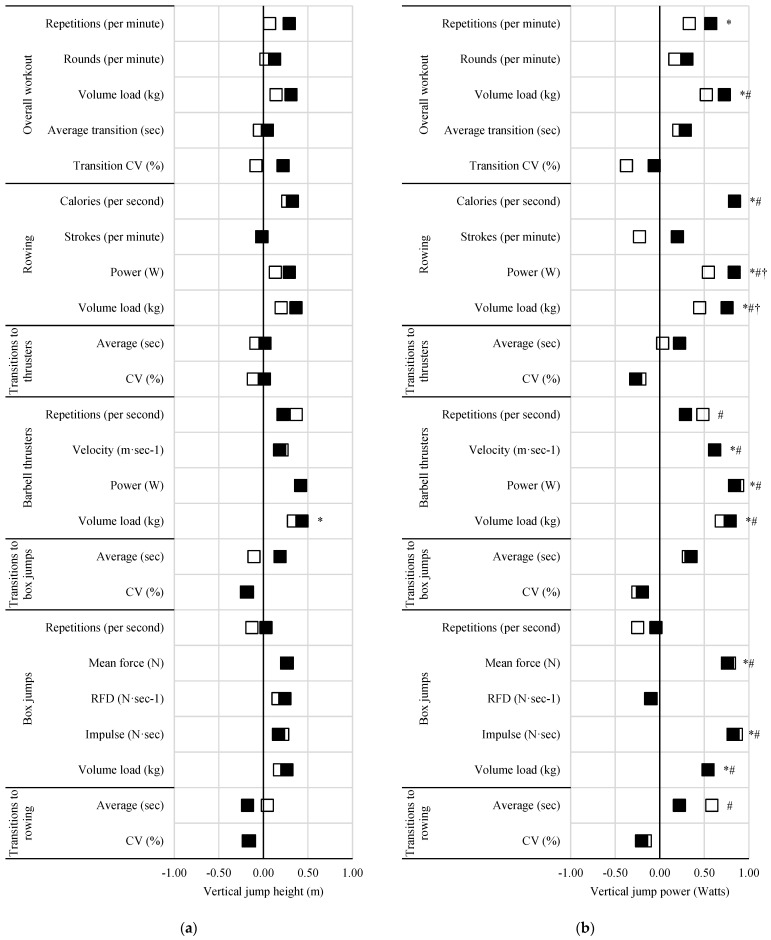
Effect of workout on relationships between measures of pacing and vertical jump (**a**) height and (**b**) power. Black squares = 5 min workout; open squares = 15 min workout; * = significant (*p* < 0.05) with 5 min workout; # = significant (*p* < 0.05) relationship with 15 min workout; † = significant (*p* < 0.05) difference between 5 and 15 min workouts.

**Figure 3:**
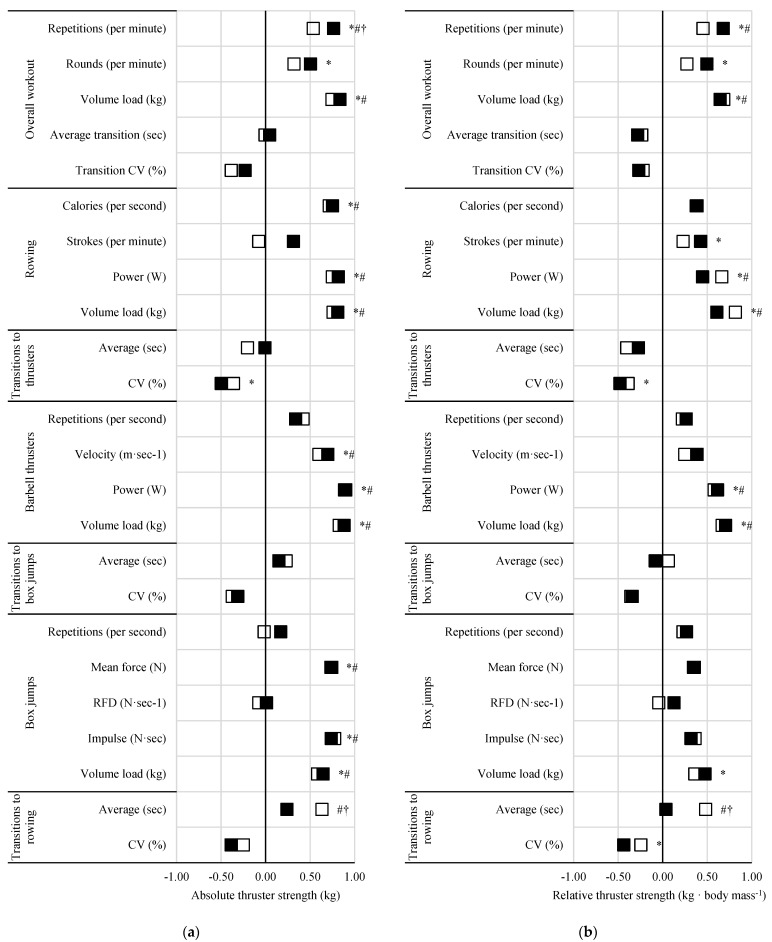
Effect of workout on relationships between measures of pacing and barbell thruster (**a**) absolute and (**b**) relative strength. Black squares = 5 min workout; open squares = 15 min workout; * = significant (*p* < 0.05) with 5 min workout; # = significant (*p* < 0.05) relationship with 15 min workout; † = significant (*p* < 0.05) difference between 5 and 15 min workouts.

**Figure 4:**
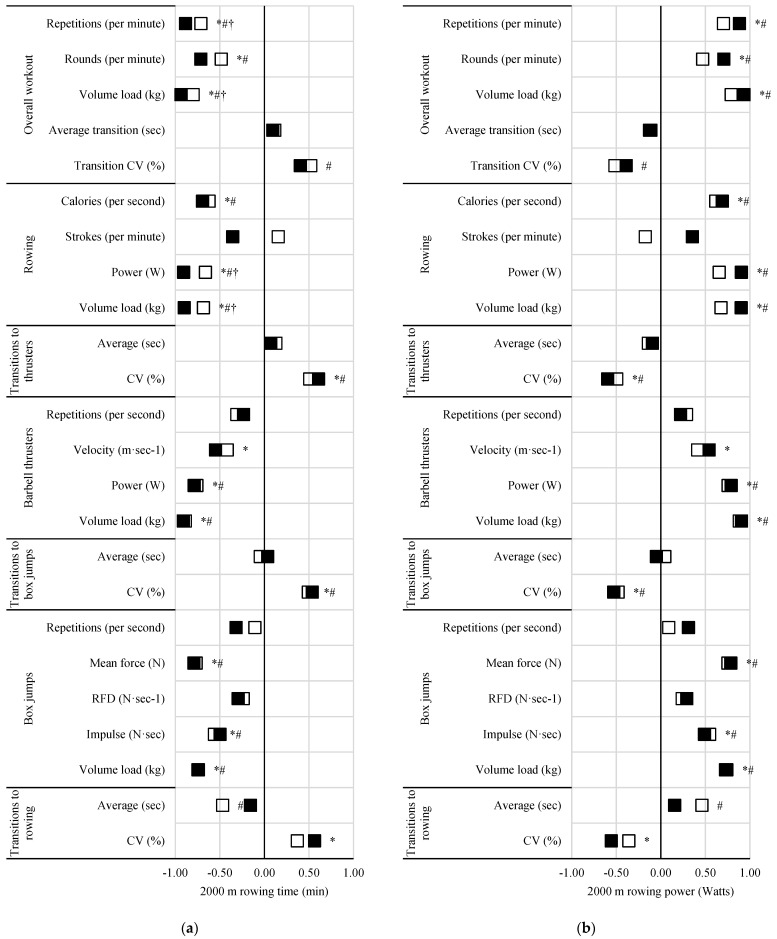
Effect of workout on relationships between measures of pacing and 2000 m rowing (**a**) time and (**b**) power. Black squares = 5 min workout; open squares = 15 min workout; * = significant (*p* < 0.05) with 5 min workout; # = significant (*p* < 0.05) relationship with 15 min workout; † = significant (*p* < 0.05) difference between 5 and 15 min workouts.

The differences amongst relationships between workout pacing and vertical jump performance, barbell thruster strength, and 2000 m rowing performance are illustrated in Figure 2, Figure 3 and Figure 4, respectively.

Positive correlations (*ρ* = 0.57 to 0.88, *p* < 0.05) were seen between 5 min repetition completion rate and vertical jump power, thruster strength (absolute and relative), and rowing power, and except for vertical jump power, these relationships were significantly stronger than those seen with the 15 min workouts (*ρ* difference = 0.18 to 0.29 to, z = 2.03 to 2.24, *p* < 0.05). Similar differences, with exceptions, were noted with 5 min round completion rate and thruster strength (absolute and relative) and 2000 m rowing performance. Rowing (power and volume load) of the 5 min workout was more strongly related to vertical jump power (*ρ* difference = 0.29 to 0.31, z = 2.04 to 2.34, *p* < 0.05) compared to that of the 15 min workout. The only relationship seen with vertical jump height was related to 5 min thruster volume load (*ρ* = 0.43, *p* = 0.044). Barbell thruster strength (absolute and relative) was negatively correlated with the CV of transitions to thrusters (*ρ* = −0.48 to −0.50, *p* < 0.05) during the 5 min but not 15 min workout. Relative barbell thruster strength was also positively correlated with 5 min rowing strokes per minute (*ρ* = 0.43, *p* = 0.047). Power and time to completion during the 2000 m rowing test were more strongly related to 5 min rowing power (*ρ* difference = 0.25, z = 2.68 to 2.70 *p* < 0.05) and volume load (*ρ* difference = 0.22 to 0.23, z = 2.23 to 2.2.45, *p* < 0.05) compared to 15 min.

Time to completion and power from the 2000 m rowing test were positively (*ρ* = 0.52, *p* = 0.017) and negatively (*ρ* = −0.52, *p* = 0.017) related to the CV of transitions during the 15 min workout, though these relationships were not statistically different from the same non-significant relationships seen during the 5 min workout. Likewise, vertical jump power was related to barbell thruster repetition completion rate in the 15 min workout (*ρ* = 0.48, *p* = 0.032) but not the 5 min workout. Vertical jump power, thruster strength (absolute and relative), and 2000 m rowing power were all positively related to transition times to rowing during the 15 min workout, with relationships to thruster absolute (*ρ* difference = 0.39, z = −2.02, *p* = 0.044) and relative strength (*ρ* difference = 0.45, z = −2.07, *p* = 0.039) being significantly stronger than those of the 5 min workouts. A negative relationship was also seen between 2000 m rowing time and transitions to rowing (total and average; *ρ* = −0.47, *p* = 0.032) for the 15 min workout only.

## 4. Discussion

Training experience, body composition, and athletic performance measures have all been known to influence HIFT workout performance [8,9,11,12,13,14,15,16,17]. However, the observed relationships have not been consistent, and an important reason for this has been that the examined workouts have shared very few similarities. Workouts have differed in structural design, exercise composition, and prescribed intensities and volumes. This study sought to overcome this limitation by examining the effect of altering a single workout detail (i.e., duration) on observed relationships. The data revealed that duration had the most pronounced effect on the relationships between athletic performance measures, 2000 m rowing performance in particular, and HIFT workout pacing and kinetics. Differential relationships to each workout duration were noted in 25.2% of paired variables, and slightly more were seen with the 5 min version. Amongst measures of training experience, most relationships had to do with the participants’ gymnastics and HIFT background. Gymnastics experience mainly affected 15 min workout performance, whereas the 5 min workout was more closely associated with HIFT experience. Measures of body composition, mainly height, body mass, and fat-free mass, were related to performance in both workouts, with about 18.4% of relationships being stronger in one duration or the other. This is the first study to examine relationships with HIFT performance when only one programming characteristic was different between workouts.

The baseline visit assessed vertical jump height and power, absolute and relative barbell thruster strength, and 2000 m rowing time and power. Since these tests were selected to match the three exercises programmed into the study workouts, it is not surprising that most relationships (~88% of workout pacing variables) were seen amongst five of the six performance variables derived from these tests. Most relationships were seen with 2000 m rowing performance, followed by absolute barbell thruster strength and vertical jump power. Indeed, 2000 m rowing power (*ρ* = 0.70–0.93) and time (*ρ* = −0.71–−0.94) were more strongly related to overall repetition completion rate and volume load completed than absolute barbell thruster strength (*ρ* = 0.54–0.84) and vertical jump power (*ρ* = 0.52–0.73). Meanwhile, body composition measures were related to pacing variables 63.2% of the time and were not as strongly related to overall performance (*ρ* = 0.43–0.78). Age and experience variables were not related to overall performance in this study. While these findings contradict the conjecture that experience, body composition, and strength are more important predictors of HIFT performance [8,9,10,13], only one other study has used a 2000 m rowing time trial as a predictor [34]. Leitao and colleagues (2021) reported a relationship to “Fran” time (*ρ* = 0.67) that was slightly better than maximal thruster strength (*ρ* = −0.61) but not as strong as thruster endurance (*ρ* = −0.82). The authors note that rowing time was also moderately related to thruster performance helps to explain its influence on a workout that did not contain rowing. Likewise, 2000 m rowing performance was related to nearly every aspect of both workouts, and rowing specifically accounted for 44–50% of total repetitions per round. Thus, it is possible that this specific workout design, regardless of duration, made rowing the single most important component.

Rowing was the most time-consuming workout component. It required the most repetitions per round, and participants completed these at the slowest rate (0.26–0.34 Calories per second) during both workouts. Participants who were less powerful on each stroke took longer to complete this exercise on each round and were even less consistent in their transitions to (5 min workout only) and from rowing (both durations). Most relationships between 2000 m rowing and workout pacing were the same between workout durations, though stronger relationships were seen with 5 min overall completion rate, volume load completed, rowing power and volume load, barbell thruster velocity, and transitions to rowing. The 5 min workout might be classified as a “sprint” within an HIFT setting due to its relatively short duration. Faster repetition completion rates and transitions are to be expected [29,30,31], and over a 5 min duration, the relative speed of these would depend on anaerobic capacity [35]. Though various indices of anaerobic capacity have been related to HIFT workout performance [8,17,34,36,37], only one previous study has examined relationships between them and a workout that required rowing [8]. Mangine and colleagues (2020) quantified anaerobic work capacity from a 3 min critical power cycling test but found that it was not related to performance in a 20 min AMRAP containing toes to bar, dumbbell hang clean and jerks, and rowing. Despite a lack of corroborating evidence, the most logical explanation is that shorter-duration workouts elevate the importance of high-intensity effort being sustained on each component. Unlike the maximal thruster and vertical jump tests, performance on the 2000 m rowing test requires both anaerobic capacity and aptitude in the modality [38,39]. Given that less time is available for breaking, any test that matches modality and reflects an ability to sustain high-intensity effort would appear to be most relevant.

Height, body mass, and fat-free mass were the most influential anthropometric measures of workout pacing, and their relationships to performance often matched the ones seen with vertical jump power and thruster strength. Taller participants with more fat-free mass, vertical jump power, and barbell thruster strength were more consistent in their transitions, took more time transitioning to rowing specifically, and were faster in completing thruster repetitions during the 15 min bout. Conversely, these traits favored 5 min overall repetition completion rate, more powerful rowing performance, and more consistent transitions to thrusters. Muscle mass, strength, and power are closely related [4,40] and are often associated with HIFT performance [9]. When paired with being taller, their combination is ideal for producing the longer and more powerful strokes needed to complete rowing sets quickly and efficiently [38,39]. That efficiency would have reduced energy expenditure during rowing sets [35] and potentially aided in readiness to perform thruster sets at a faster completion rate. These attributes were often relevant to the same pacing aspects of the 15 min bout, but they were not as important. Nevertheless, being more powerful and efficient in rowing might explain why greater patience and consistency were employed when progressing to and from rowing, respectively. It is possible that the more powerful rowers in this study chose a more conservative and sustainable rowing pace, knowing each round would still be completed in a timely fashion and leave them better prepared for barbell thrusters. Of all the traditional aerobic modalities, rowing has been prescribed the most in HIFT competition [27,41]. These data suggest that a better understanding of the direct and indirect effects rowing ability has on performance in various HIFT-style workouts is warranted.

The nature of past training experiences has been shown to be more important to HIFT performance than just possessing several years of training experience [13]. Although this study was not designed to expand on that topic, the data do suggest that the type of experience matters. Like a previous report [8], resistance training experience was neither related to overall performance nor any individual aspect of the 5 and 15 min workout pacing. Instead, more gymnastics experience was associated with shorter, more consistent transitions during the 15 min bout, especially when heading into box jumps. Meanwhile, greater HIFT experience led to more time spent transitioning to rowing and a faster barbell thruster pace during the 5 min workout. With this being the first study to simultaneously examine the influence of these three types of training experience (i.e., resistance training, gymnastics, and HIFT), it is difficult to explain these results based on past evidence. Still, it is reasonable to speculate that resistance training experience may not have been important because the workout only included one moderate intensity lift. At roughly 50% of the participants’ maximal strength, six barbell thruster repetitions per round were not likely to have presented a challenge by themselves [3,35]. Rather, the challenge would have been to maintain a consistent repetition completion rate within the context of an AMRAP design, which is a skill more likely to have been developed through HIFT experience.

The influence of gymnastics over HIFT experience is interesting but may have been a coincidence. This study was not designed or powered to specifically examine the influence of different types of experience, and so, only one-third of participants possessed any gymnastics experience. Since these individuals also averaged the same amount of resistance training and HIFT experience as all other participants, it is possible that their gymnastics experience acted as a modifier. In addition to strength and power, gymnastics requires a great deal of balance and coordination [42], and these attributes may have led to a more efficient execution of transition strategy between exercises and with cycling box jump repetitions. The influence of experience on HIFT performance has been given limited attention in research [8,13,31,37], and the present results suggest there are several remaining questions in need of answers before this characteristic’s position of hierarchical importance can be known.

## 5. Conclusions

Sport-specific training is only possible when the needs of the sport are known. Consistent with its definition [5,6], existing research on HIFT suggests several needs [8,9,11,12,13,14,15,16,17]. However, specific needs vary between workouts and are difficult to predict, aside from the obvious (e.g., barbell thruster strength being relevant to a workout containing thrusters). Previously examined workouts have differed in structural design, exercise composition, repetition scheme, assigned loads, and intended duration. Each represents its own factor, and with so many uncontrolled factors being present, generalized conclusions are difficult without significantly more research involving several more HIFT-style workouts or research that isolates single factors for examination. Thus, the present study opted for efficiency by examining how duration alone might influence relevant traits. The data confirmed several expected relationships but also revealed their differential influence when the same AMRAP circuit was completed over a longer duration. Performance measures appeared to be more applicable to the 5 min workout, whereas the relevance of body composition was more evenly split. When variables were more strongly related to the 15 min workout (e.g., experience and body composition measures), they most often had to do with transition strategy. These observations suggest that athletic traits may have a more direct impact on the effort employed for each exercise programmed for short-duration HIFT workouts. These traits are still relevant to longer-duration workouts, but experience and body composition seem to modulate what happens between exercises. Therefore, while coaches and athletes should still train for healthy body composition and improvements in all relevant physiological traits, they must understand that such improvements will not always impact performance the same way.

Of course, several limitations of this study must be acknowledged. All the men and women who volunteered for this study possessed at least 2 years of HIFT experience but had not yet advanced beyond the opening round of any official international HIFT competition. It cannot be assumed that duration would impact less experienced or more advanced athletes in the same way. It is also unknown whether a completely different circuit, or a rearranged exercise prescription, would be affected similarly by duration. The present workout designed rowing to be the most time-consuming exercise and to account for nearly half of all repetitions. A redistribution of repetition assignments might have led to different results. It is interesting to note that significant differences were observed between the correlation coefficients of variables that were not significantly related to either workout duration. It is possible that these instances were representative of real differences that could not be observed due to a lack of statistical power. As the first of its kind, and being an exploratory study, a great deal of guesswork was needed to determine whether the present sample would be sufficient to avoid a type II error. Ultimately, the sample was sufficient to observe several relationships and differences, and a larger sample might have only revealed significant but trivial relationships. Future investigations should continue to isolate factors before expanding to comparisons that involve multiple programming differences. By continuing to isolate factors in different types of HIFT workouts (a standard classification system is still needed) with samples involving a wider range of tiered participants, a better understanding of how each factor affects physiology is possible. Without understanding how each programming decision affects the training stress, purposeful programming will remain elusive.

## Figures and Tables

**Table 1:** Participant baseline characteristics.

	Mean ± SD	(Range)
Experience (years)		
Age	29.3 ± 7.1	(18.4–42.9)
Resistance training	11.1 ± 5.8	(5.0–30.0)
Gymnastics	1.6 ± 2.5	(0.0–7.0)
High-intensity functional training	5.9 ± 2.8	(2.0–12.0)
Body composition		
Height (m)	171 ± 7	(159–191)
Body mass (kg)	80.5 ± 15.6	(53.2–108.3)
Fat mass (kg)	14.6 ± 6.3	(5.1–27.7)
Fat-free mass (kg)	65.9 ± 13.5	(44.0–92.2)
Body fat percentage (%)	18.1 ± 6.5	(5.6–28.5)
Vertical jump performance		
Height (m)	0.49 ± 0.15	(0.28–0.73)
Estimated average power (Watts)	1564 ± 479	(669–2292)
Estimated barbell thruster strength		
Absolute (kg)	75.1 ± 21.9	(43.2–123.6)
Relative (kg body mass^−1^)	0.93 ± 0.17	(0.66–1.23)
2000 m rowing performance		
Completion time (minutes)	7.95 ± 0.71	(6.73–9.26)
Average power (Watts)	216 ± 56	(131–340)

**Table 2:** Workout performances.

	5 min		15 min	
	Mean ± SD	(Range)	Mean ± SD	(Range)
Overall performance				
Repetitions (per minute)	15.5 ± 2.5	(11.0–21.6)	12.4 ± 2	(8.9–17.1)
Rounds (per minute)	0.91 ± 0.12	(0.69–1.20)	0.72 ± 0.11	(0.54–0.95)
Volume load (kg)	5970 ± 1514	(3396–9389)	13,032 ± 3253	(7285–21,235)
Average transition time (seconds)	19.06 ± 2.53	(13.80–24.60)	22.13 ± 3.32	(17.33–29.40)
Transition time coefficient of variation (%)	0.13 ± 0.06	(0.02–0.26)	0.22 ± 0.05	(0.08–0.28)
Rowing performance				
Calories (per second)	0.38 ± 0.07	(0.23–0.47)	0.26 ± 0.05	(0.15–0.36)
Strokes (per minute)	31.4 ± 4.3	(23.2–40.6)	26.1 ± 4.1	(13.5–34.6)
Average power (Watts)	270 ± 75	(157–409)	183 ± 54	(65–287)
Volume load (kg)	3181 ± 757	(1958–4521)	6226 ± 1845	(2200–10,503)
Transitions to barbell thrusters				
Average time (seconds)	8.26 ± 1.30	(6.60–11.40)	9.22 ± 1.40	(7.00–12.80)
Coefficient of variation (%)	0.31 ± 0.19	(0.05–0.83)	0.59 ± 0.19	(0.16–0.98)
Barbell thrusters performance				
Repetitions (per second)	0.46 ± 0.08	(0.28–0.63)	0.43 ± 0.07	(0.30–0.55)
Average velocity (m·sec^−1^)	1.18 ± 0.18	(0.73–1.58)	1.18 ± 0.17	(0.74–1.48)
Average power (Watts)	434 ± 124	(211–666)	430 ± 117	(213–624)
Volume load (kg)	988 ± 276	(531–1551)	2372 ± 557	(1415–3620)
Transitions to box jumps				
Average time (seconds)	4.14 ± 0.94	(2.20–7.20)	5.22 ± 1.76	(3.20–10.13)
Coefficient of variation (%)	0.42 ± 0.21	(0.14–0.82)	0.65 ± 0.19	(0.20–0.99)
Box jumps performance				
Repetitions (per second)	0.45 ± 0.06	(0.31–0.58)	0.41 ± 0.07	(0.26–0.54)
Mean force (N)	1380 ± 257	(842–1807)	1352 ± 261	(869–1777)
Average RFD (N·sec^−1^)	10,928 ± 4419	(3924–20,396)	10,897 ± 4669	(4693–22,377)
Average impulse (N·sec)	304 ± 118	(162–599)	303 ± 124	(89–576)
Volume load (kg)	1801 ± 590	(773–3316)	4435 ± 1374	(2471–7112)
Transitions to rowing				
Average time (seconds)	6.50 ± 1.19	(4.80–10.00)	7.32 ± 1.17	(5.27–10.60)
Coefficient of variation (%)	0.44 ± 0.23	(0.06–0.83)	0.63 ± 0.18	(0.20–0.96)

**Table 3:** Effect of workout duration on relationships between measures of pacing and age and experience.

			Experience (Years)					
	Age (years)		Resistance Training		Gymnastics		HIFT	
	5 min	15 min	5 min	15 min	5 min	15 min	5 min	15 min
Overall workout								
Repetitions (per minute)	−0.24	−0.27	0.05	−0.14	0.01	0.33 †	−0.25	0.17 †
Rounds (per minute)	−0.22	−0.25	−0.05	−0.17	0.07	0.30	−0.05	0.44 †
Volume load (kg)	−0.28	−0.18	0.04	0.00	−0.06	0.08	−0.21	−0.01
Average transition (sec)	0.05	0.19	0.04	−0.04	−0.12	−0.50 #	0.49 *	0.01 †
Transition CV (%)	0.09	0.33	0.05	0.28	−0.12	0.03	−0.16	−0.11
Rowing								
Calories (per second)	−0.26	−0.23	0.11	0.04	−0.16	−0.28	−0.21	−0.30
Strokes (per minute)	−0.10	0.27	0.26	0.12	0.29	0.04	−0.13	0.34
Power (W)	−0.23	−0.17	0.07	0.10	−0.13	−0.14	−0.18	−0.07
Volume load (kg)	−0.27	−0.19	0.12	0.10	−0.02	0.06	−0.30	−0.08
Transitions to thrusters								
Average (sec)	−0.21	0.04	−0.18	−0.27	−0.18	−0.32	0.39	−0.10 †
CV (%)	0.23	0.49 #	0.14	0.21	0.02	−0.46 #†	−0.01	−0.22
Barbell thrusters								
Repetitions (per second)	0.13	0.12	0.27	0.13	−0.05	−0.08	0.45 *	0.38
Velocity (m·sec^−1^)	−0.01	0.01	0.16	0.20	−0.29	−0.33	0.11	0.23
Power (W)	−0.18	−0.19	0.16	0.15	−0.20	−0.22	−0.23	−0.15
Volume load (kg)	−0.32	−0.33	0.11	0.00	−0.06	0.17 †	−0.26	−0.11
Transitions to box jumps								
Average (sec)	0.40	0.26	0.41	0.08	−0.18	−0.48 #	0.20	−0.05
CV (%)	0.01	0.36	−0.05	0.14	0.10	−0.29	0.01	−0.45 #
Box jumps								
Repetitions (per second)	−0.20	−0.21	−0.24	−0.13	0.27	0.50 #	0.06	0.37
Mean force (N)	−0.07	−0.13	0.11	0.01	−0.22	−0.11	0.13	0.15
RFD (N·sec^−1^)	−0.14	−0.11	−0.26	−0.21	−0.26	−0.14	−0.03	−0.09
Impulse (N·sec)	0.03	−0.03	0.35	0.24	0.01	−0.13 †	0.05	0.12
Volume load (kg)	−0.12	−0.24	0.07	−0.14	−0.13	0.01	−0.01	0.22
Transitions to rowing								
Average (sec)	0.11	−0.16	0.23	0.23	0.25	−0.08	0.57 *	0.11 †
CV (%)	0.22	0.36	0.10	0.16	0.12	−0.35 †	−0.13	−0.38

* = Significant (*p* < 0.05) with 5 min workout; # = significant (*p* < 0.05) relationship with 15 min workout; † = significant (*p* < 0.05) difference between 5 and 15 min workouts.

**Table 4:** Effect of workout duration on relationships between measures of pacing and body composition.

	Height (m)		Body Mass (kg)		Fat Mass (kg)		Fat-Free Mass (kg)		Body Fat (%)	
	5 min	15 min	5 min	15 min	5 min	15 min	5 min	15 min	5 min	15 min
Overall workout										
Repetitions (per minute)	0.51 *	0.43 #	0.55 *	0.46 #	−0.06	−0.05	0.64 *	0.52 #	−0.37	−0.29
Rounds (per minute)	0.35	0.37	0.37	0.32	−0.08	0.01	0.45 *	0.34	−0.31	−0.15
Volume load (kg)	0.64 *	0.49 #	0.68 *	0.58 #	−0.01	0.00	0.78 *	0.63 #	−0.37	−0.26
Average transition (sec)	0.33	0.22	0.38	0.20	0.33	0.10	0.27	0.18	0.24	0.03
Transition CV (%)	−0.19	−0.40	−0.14	−0.47 #	0.08	−0.02	−0.22	−0.50 #	0.22	0.17
Rowing										
Calories (per second)	0.67 *	0.66 #	0.72 *	0.67 #	0.05	0.03	0.84 *	0.78 #	−0.27	−0.28
Strokes (per minute)	0.09	−0.38	0.06	−0.22	−0.18	−0.18	0.21	−0.20	−0.34	−0.04
Power (W)	0.79 *	0.53 #†	0.82 *	0.56 #†	0.10	−0.04	0.88 *	0.64 #†	−0.29	−0.23
Volume load (kg)	0.63 *	0.39	0.65 *	0.43 #	0.00	−0.14	0.75 *	0.54 #	−0.37	−0.33
Transitions to thrusters										
Average (sec)	0.39	0.12	0.24	0.06	0.22	−0.03	0.20	0.06	0.12	−0.07
CV (%)	−0.34	−0.33	−0.31	−0.23	0.03	0.22	−0.41	−0.37	0.23	0.42
Barbell thrusters										
Repetitions (per second)	0.30	0.37	0.41	0.54 #	0.21	0.25	0.32	0.48 #	0.03	0.02
Velocity (m·sec^−1^)	0.70 *	0.72 #	0.67 *	0.67 #	−0.02	0.04	0.74 *	0.68 #	−0.32	−0.22
Power (W)	0.70 *	0.77 #†	0.75 *	0.77 #	−0.07	−0.02	0.87 *	0.87 #	−0.43	−0.39
Volume load (kg)	0.63 *	0.60 #	0.66 *	0.65 #	−0.03	−0.06	0.79 *	0.76 #	−0.40	−0.41
Transitions to box jumps										
Average (sec)	0.17	0.24	0.31	0.32	0.14	−0.02	0.28	0.35	0.03	−0.12
CV (%)	−0.26	−0.36	−0.23	−0.28	−0.01	0.08	−0.23	−0.38	0.12	0.24
Box jumps										
Repetitions (per second).	0.03	−0.11	0.09	−0.22	−0.29	−0.54 #	0.19	−0.03	−0.41	−0.53 #
Mean force (N)	0.75 *	0.75 #	0.85 *	0.88 #	0.22	0.31	0.84 *	0.85 #	−0.11	−0.06
RFD (N·sec^−1^)	−0.04	−0.01	−0.03	−0.02	−0.04	0.03	−0.03	−0.05	−0.04	0.04
Impulse (N·sec)	0.71 *	0.74 #	0.83 *	0.85 #	0.28	0.28	0.79 *	0.83 #	−0.06	−0.07
Volume load (kg)	0.50 *	0.59 #	0.63 *	0.67 #	0.08	0.18	0.64 *	0.65 #	−0.18	−0.13
Transitions to rowing										
Average (sec)	0.31	0.61 #	0.44 *	0.51 #	−0.01	−0.18	0.41	0.62 #	−0.11	−0.39
CV (%)	−0.28	−0.28	−0.27	−0.19	0.08	0.21	−0.32	−0.31	0.23	0.32

* = Significant (*p* < 0.05) with 5 min workout; # = significant (*p* < 0.05) relationship with 15 min workout; † = significant (*p* < 0.05) difference between 5 and 15 min workouts.

## Data Availability

Data will be made available upon an emailed request to the corresponding author.

## Abbreviations

The following abbreviations are used in this manuscript:

AMRAP	As many repetitions as possible
BF%	Body fat percentage
CSCS	Certified strength and conditioning specialist
CV	Coefficient of variation
HIFT	High-intensity functional training
RFD	Rate of force development
RM	Repetition maximum
